# Variation in HIV Transmission Behaviors Among People Who Use Drugs in Rural US Communities

**DOI:** 10.1001/jamanetworkopen.2023.30225

**Published:** 2023-08-21

**Authors:** Wiley D. Jenkins, Samuel R. Friedman, Christopher B. Hurt, P. Todd Korthuis, Judith Feinberg, Lizbeth M. Del Toro-Mejias, Suzan Walters, David W. Seal, Rob J. Fredericksen, Ryan Westergaard, William C. Miller, Vivian F. Go, John Schneider, Mihai Giurcanu

**Affiliations:** 1 Department of Population Science and Policy, Southern Illinois University School of Medicine, Springfield; 2 Department of Population Health, New York University Grossman School of Medicine, New York; 3 Institute for Global Health and Infectious Diseases, University of North Carolina at Chapel Hill; 4 Addiction Medicine Section, Oregon Health and Science University, Portland; 5 West Virginia University School of Medicine, Morgantown; 6 Office of Research, University of Massachusetts Chan Medical School-Baystate, Springfield; 7 School of Global Public Health, New York University, New York, New York; 8 Tulane University School of Public Health and Tropical Medicine, New Orleans, Louisiana; 9 Department of Medicine, University of Washington, Seattle; 10 School of Medicine and Public Health, University of Wisconsin, Madison; 11 Division of Epidemiology, College of Public Health, The Ohio State University, Columbus; 12 Department of Health Behavior, Gillings School of Global Public Health, University of North Carolina at Chapel Hill; 13 Biological Sciences Division, University of Chicago, Chicago, Illinois; 14 Department of Public Health Sciences, University of Chicago, Chicago, Illinois

## Abstract

**Importance:**

People who use drugs (PWUD) continue to be at risk of HIV infection, but the frequency and distribution of transmission-associated behaviors within various rural communities is poorly understood.

**Objective:**

To examine the association of characteristics of rural PWUD with HIV transmission behaviors.

**Design, Setting, and Participants:**

In this cross-sectional study, surveys of PWUD in rural communities in 10 states (Illinois, Kentucky, New Hampshire, Massachusetts, North Carolina, Ohio, Oregon, Vermont, West Virginia, and Wisconsin) were collected January 2018 through March 2020 and analyzed August through December 2022. A chain-referral sampling strategy identified convenience sample seeds who referred others who used drugs. Rural PWUD who reported any past 30-day injection drug use or noninjection opioid use “to get high” were included.

**Exposures:**

Individual characteristics, including age, race, gender identity, sexual orientation, partnership status, drug of choice, and location, were collected.

**Main Outcomes and Measures:**

Past 30-day frequency of behaviors associated with HIV transmission, including drug injection, syringe sharing, opposite- and same-gender partners, transactional sex, and condomless sex, was assessed.

**Results:**

Of 3048 rural PWUD (mean [SD] age, 36.1 [10.3] years; 225 American Indian [7.4%], 96 Black [3.2%], and 2576 White [84.5%] among 3045 with responses; and 1737 men [57.0%] among 3046 with responses), most participants were heterosexual (1771 individuals [86.8%] among 2040 with responses) and single (1974 individuals [68.6%] among 2879 with responses). Opioids and stimulants were reported as drug of choice by 1636 individuals (53.9%) and 1258 individuals (41.5%), respectively, among 3033 individuals with responses. Most participants reported recent injection (2587 of 3046 individuals [84.9%] with responses) and condomless sex (1406 of 1757 individuals [80.0%] with responses), among whom 904 of 1391 individuals (65.0%) with responses indicated that it occurred with people who inject drugs. Syringe sharing (1016 of 2433 individuals [41.8%] with responses) and transactional sex (230 of 1799 individuals [12.8%] with responses) were reported less frequently. All characteristics and behaviors, except the number of men partners reported by women, varied significantly across locations (eg, mean [SD] age ranged from 34.5 [10.0] years in Wisconsin to 39.7 [11.0] years in Illinois; *P* < .001). In multivariable modeling, younger age (adjusted odds ratio [aOR] for ages 15-33 vs ≥34 years, 1.36; 95% CI, 1.08-1.72) and being single (aOR, 1.37; 95% CI, 1.08-1.74) were associated with recent injection; younger age (aOR, 1.49; 95% CI, 1.20-1.85) and bisexual orientation (aOR vs heterosexual orientation, 2.27; 95% CI, 1.60-3.23) with syringe sharing; gender identity as a woman (aOR vs gender identity as a man, 1.46; 95% CI, 1.01-2.12), bisexual orientation (aOR vs heterosexual orientation, 2.59; 95% CI, 1.67-4.03), and being single (aOR, 1.71; 95% CI, 1.15-2.55) with transactional sex; and bisexual orientation (aOR vs heterosexual orientation, 1.60; 95% CI, 1.04-2.46) and stimulants as the drug of choice (aOR vs opioids, 1.45; 95 CI, 1.09-1.93) with condomless sex with someone who injects drugs.

**Conclusions and Relevance:**

This study found that behaviors associated with HIV transmission were common and varied across communities. These findings suggest that interventions to reduce HIV risk among rural PWUD may need to be tailored to locally relevant factors.

## Key Points

**Question:**

What are the frequency and distribution of HIV transmission behaviors among people who use drugs (PWUD) who live in rural areas?

**Findings:**

In this cross-sectional study of 3048 PWUD living in rural areas of the US, substantial proportions of individuals reported drug injection (84.9%), sharing syringes (41.8%), condomless sex (80.0%), and transactional sex (7.5%). Study sites had significant variation in participant characteristics (eg, race and sexual orientation) and HIV transmission behaviors, and some participant characteristics (eg, age and sexual orientation) were more frequently associated with transmission behaviors.

**Meaning:**

These findings suggest that “rural PWUD” may not be a homogeneous construct for which universal interventions may be equally effective across populations and regions and that identification of local characteristics and behaviors may be needed for effective measures to be developed and implemented.

## Teaser

This cross-sectional study investigates the association of factors among people who use drugs in rural communities with HIV transmission–associated behaviors.

## Introduction

After years of declining incidence, cases of HIV attributable to injection drug use are increasing in the US. A yearlong outbreak associated with sharing equipment to inject oxymorphone was associated with 215 diagnoses in rural Scott County, Indiana.^1,2^ A more recent HIV outbreak occurred in West Virginia among 85 persons in a network of people who inject drugs.^3^ These outbreaks reflect a trend: the transmission of HIV among people who inject drugs is shifting out of urban centers and into the rural US. This is explicitly highlighted in a Centers for Disease Control and Prevention risk assessment using community-level factors associated with hepatitis C virus diagnosis (a proxy for HIV) that identified 220 overwhelmingly rural counties susceptible to a similar HIV outbreak.^4^ Furthermore, 7 US states were described as having sustained rural transmission (ie, ≥10% of cases from rural areas) in the Ending the HIV Epidemic in the US plan. Southern states now account for more than 50% of new HIV cases, with a large proportion (24%) diagnosed in suburban and rural areas.^5,6^

HIV remains largely driven by sexual transmission, with 67.8% of new diagnoses in the US among men who have sex with men (MSM) and 6.7% associated with heterosexual sex.^7,8^ The confluence of HIV transmission risk and drug use was observed in data showing that past-year injection among men who have sex with men varied by HIV status (1.9% for HIV-negative vs 5.2% for HIV-positive statuses) and that people who use drugs (PWUD) frequently report condomless sex.^9,10^ Age also remains an important factor given that younger individuals more frequently report behaviors associated with transmission, HIV diagnoses, and substance use disorders.^7,11,12^ However, much of the data is drawn from large cities, and risk may substantially differ in nonmetropolitan communities, where some drug use rates are known to be higher and sexual risks may be greater.^13,14,15^

The HIV risk environment for rural PWUD remains incompletely characterized. Some data indicate that rural PWUD may be more likely to initiate drug use at an earlier age, begin injecting earlier, and engage in polysubstance use, especially combinations of opioids with stimulants.^16^ Sexual transmission–related risk has received less attention in rural communities but remains significant.^17^ In a 2017 HIV outbreak in rural West Virginia, for example, cases were associated with male-to-male sexual contact (34 cases [60%]), injection drug use (5 cases [9%]), male-to-male sexual contact and injection drug use (3 cases [5%]), and heterosexual contact (2 cases [4%]).^18^ Modeling studies suggest that implementing syringe services programs may be associated with effective reduction of HIV cases.^2,19^ Fortunately, these programs have increased considerably since 2015, including in rural areas.^20^ While sexual risk reduction is a common component of syringe services programs and other harm-reduction programs, little is known about sexual transmission behaviors among rural PWUD.^21,22,23^

The rural experience of HIV transmission behaviors associated with drug use and sexual activity often occurs in the context of health care environments with very limited resources for prevention or treatment.^24^ This is associated with the relative scarcity of clinicians, distance to health care facilities, and near-total absence of public transportation and is compounded by the lack of HIV-specific programs and resources.^17,25,26,27,28,29^ Fewer rural counties have any HIV-related services available (75% vs 91% of urban counties), and multiple studies^30,31,32,33,34^ have noted similar rural-urban differences for direct services, rapid testing, prevention education, and preexposure prophylaxis availability. These limitations are particularly exacerbated among PWUD whose frequent stigmatization in health care environments limits their desire and ability to access routine and preventive care.^35^

Data suggest that HIV transmission behaviors vary across geography and populations, further complicating mitigation strategies. There is substantial and significant variation in cocaine, heroin, and methamphetamine use and injection rates across states; moreover, drug use and injection may also significantly vary by race and ethnicity, age, sexual orientation, and gender.^36,37,38,39,40,41,42,43,44,45,46^ Other HIV risk behaviors vary by race (eg, condom use among men who have sex with men), gender identity (eg, past-year and lifetime number of partners and drug use disorder diagnosis), and sexual orientation (eg, transactional sex).^47,48,49,50,51^ Among rural PWUD, transactional sex has been shown to vary by gender, sexual orientation, partnership status, and injection behavior.^52^ Overall, existing data indicate that the HIV risk environment for rural PWUD is multifaceted and highly variable. The objective of this work was to describe the distribution of HIV transmission–associated behaviors among rural PWUD and how these may vary by individual-level characteristic and location.

## Methods

### Study Design, Setting, and Participants

In this cross-sectional study, we conducted a survey from January 2018 through March 2020 of PWUD in rural counties with high overdose rates across 8 project areas (sites) in 10 states participating in the Rural Opioid Initiative (ROI): Illinois; Kentucky; North Carolina; Massachusetts, New Hampshire, and Vermont [New England]; Ohio; Oregon; West Virginia; and Wisconsin). All sites obtained local institutional review board approval for research activities and data sharing (eMethods in Supplement 1). Participants provided informed consent. A full description of the ROI structure and operations is described elsewhere, and a detailed description of the ROI project sites, its work, and its publications can be found on the ROI Research Consortium Studies website.^37,53^ We reported results in accordance with Strengthening the Reporting of Observational Studies in Epidemiology (STROBE) reporting guideline for observational studies.

Individuals were eligible for inclusion if they lived in the study area, reported any past 30-day injection drug use or noninjection opioid use “to get high,” were able to communicate in English, and met site-specific age criteria (ages ≥15 years at 2 sites and ≥18 years at 6 sites). Participants were recruited between January 2018 and March 2020 using a modified chain referral based on respondent-driven sampling.^54,55^ Sites enrolled “seeds” who met eligibility criteria and agreed to recruit 3 to 6 members of their social network who may be eligible. Participants received $10 to $20 per successfully enrolled peer and $40 to $60 for completion of study procedures.

### Data Sources and Measurement

After recruitment and informed consent, participants were offered an HIV test (rapid test in 7 sites and standard testing in 1 site) and completed a standardized, structured questionnaire collected by audio, computer-assisted self-interview at 5 sites; computer-assisted personal interview at 2 sites (REDCap and Questionnaire Development System); and computer-assisted self-interview at 1 site (Qualtrics) (see software version numbers in eMethods in Supplement 1).^56,57,58^ The questionnaire assessed participant self-reported characteristics and behaviors. Data were transferred to the ROI Data Coordinating Center at the University of Washington for quality review and collation of a national analytic data set.

### Variables

Participant characteristics included site and the following self-reported items plausibly associated with differences in drug use and sexual activity: age, race, gender identity, sexual orientation, partnership status, and drug of choice. In this analysis, age was operationalized as younger (aged 15-33 years) vs older (aged ≥34 years) and as a mean. Other variables were consolidated based on frequency, including race (American Indian, Black, White, and other), gender identity (men, women, and other), sexual orientation (heterosexual, gay or lesbian, bisexual, and other), partnership status (partnered and unpartnered), and drug of choice (opioids, stimulants, and other). Categories for race in the survey were African; African American or Black; Alaskan Native; American Indian; Asian, Pacific Islander, Native Hawaiian; White; mixed race; and other. Categories other than American Indian, Black, and White were combined as “other” owing to small sample sizes. We selected behaviors that were assessed for the past 30 days and were plausibly associated with HIV transmission, including those associated with drug use (injection and syringe sharing) and sex (vaginal or anal sex, number of women and men partners, and sex that was transactional [trading vaginal or anal sex for drugs, money, housing, or other needed things], condomless, or condomless with someone who injects drugs). Behaviors were dichotomized into zero vs any occurrences (0 vs ≥1 occurrences) except for opposite-sex partners, which were dichotomized to zero or 1 vs 2 or more occurrences (0-1 vs ≥2 occurrences). Some data were not routinely collected by all sites, including sexual orientation (Wisconsin), and past 30-day injection drug use was required eligibility for Wisconsin participants.

### Addressing Potential Bias

Recent theoretical and empirical work has assessed strengths and weaknesses of respondent-driven sampling.^59,60,61^ This work has emphasized the importance of careful selection of seeds from diverse sources and sufficient iterative rounds of recruitment to penetrate further reaches of the larger social-networked population being studied. While study sites used respondent-driven sampling primarily as an effective means for participant recruitment, criteria required for such sampling to be generally representative of the rural PWUD population (eg, seed selection and subsequent recruitment waves) were not met. Thus, the sample for this analysis reflects a convenience sample with biases associated with a lack of systematic sample generation.

### Statistical Analysis

Data were analyzed August through December 2022. We calculated summary statistics for participant characteristic variables overall and stratified by site to assess the variability and degree of association between participant characteristics and sites (Table 1). Between-variable associations were tested using the *F* test for the continuous variable (age) and Pearson χ^2^ test for categorical variables. The distributions of HIV-transmission behavior (ie, dependent) variables were examined overall and stratified by participant characteristic, and associations were investigated using the χ^2^ or Fisher exact test (Table 2, Table 3, and Table 4). Odds ratios (ORs) with 95% CIs of each behavior relative to each characteristic were calculated (eTable in Supplement 1). Finally, each behavior was further analyzed in a logistic regression model adjusting for all characteristics to determine adjusted ORs (aORs; Table 5). As we were exploring the association of each characteristic with each behavior, we purposefully explored each bivariate comparison and included all exposures in the full model for each outcome. Because our sample was a convenience sample, all *P* values are used as a heuristic given that they were not based on a probability sample. Significance was assumed at a *P* value < .05, and all comparisons were 2-sided. Significance for ORs and aORs was assumed when CIs did not include 1.0. Analyses were performed using SPSS statistical software version 29.0.1.0 for Windows (IBM).

**Table 1.** Participant Characteristics by Site

Characteristic	Responses, No.	Participants, No. (%)									*P* value
		Overall (N = 3048)	IL (n = 173)	KY (n = 388)	NC (n = 350)	NE (n = 589)	OH (n = 258)	OR (n = 174)	WI (n = 991)	WV (n = 175)	
Age, mean (SD), y	3048	36.1 (10.3)	39.7 (11.0)	36.0 (8.9)	35.0 (11.1)	35.8 (10.4)	39.5 (9.9)	37.0 (10.4)	34.5 (10.0)	38.6 (9.8)	<.001
Race											
American Indian	3045	225 (7.4)	3 (1.7)	1 (0.3)	85 (24.4)	9 (1.5)	5 (1.9)	9 (5.2)	111 (11.2)	2 (1.1)	<.001
Black		96 (3.2)	18 (10.4)	2 (0.6)	5 (1.4)	7 (1.2)	13 (5.0)	3 (1.7)	35 (3.5)	13 (7.4)	
White		2576 (84.6)	148 (85.5)	331 (97.9)	242 (69.3)	533 (90.5)	231 (89.5)	145 (83.3)	792 (80.1)	154 (88.0)	
Other^a^		148 (4.9)	4 (2.3)	4 (1.2)	17 (4.9)	40 (6.8)	9 (3.5)	17 (9.8)	51 (5.2)	6 (3.4)	
Gender identity											
Men	3046	1737 (57.0)	100 (57.8)	193 (57.1)	182 (52.0)	343 (58.2)	127 (49.2)	99 (56.9)	584 (58.9)	109 (62.3)	.02
Women		1293 (42.2)	73 (42.2)	144 (42.6)	168 (48.0)	243 (41.3)	130 (50.4)	75 (43.1)	394 (39.8)	66 (37.7)	
Transgender or other		16 (0.5)	0 (0.0)	1 (0.3)	0 (0.0)	3 (0.5)	1 (0.4)	0 (0.0)	11 (1.1)	0 (0.0)	
Sexual orientation^b^											
Heterosexual	2040	1771 (86.8)	142 (83.0)	315 (93.2)	299 (86.4)	485 (83.0)	225 (87.9)	151 (88.3)	NA	154 (88.5)	.002
Gay or lesbian		40 (2.0)	8 (4.7)	7 (2.1)	8 (2.3)	8 (1.4)	1 (0.4)	4 (2.3)	NA	4 (2.3)	
Bisexual		219 (10.7)	20 (11.7)	16 (4.7)	38 (11.0)	86 (14.7)	28 (10.9)	15 (8.8)	NA	16 (9.2)	
Other		10 (0.5)	1 (0.6)	0 (0.0)	1 (0.3)	5 (0.9)	2 (0.8)	1 (0.6)	NA	0 (0.0)	
Partnership status											
Partnered	2879	905 (31.4)	42 (24.7)	137 (40.7)	104 (31.1)	147 (26.7)	79 (31.5)	40 (23.8)	315 (35.1)	41 (24.1)	<.001
Unpartnered		1974 (68.6)	128 (75.3)	200 (59.3)	230 (68.9)	404 (73.3)	172 (68.5)	128 (76.2)	583 (64.9)	129 (75.9)	
Drug of choice											
Opioids	3033	1636 (53.9)	81 (46.8)	206 (60.9)	171 (48.9)	450 (76.4)	183 (70.9)	78 (44.8)	362 (37.1)	105 (60.0)	<.001
Stimulants		1258 (41.5)	86 (49.7)	115 (34.0)	172 (49.1)	118 (20.0)	69 (26.7)	92 (52.9)	541 (55.4)	65 (37.1)	
Other		139 (4.6)	6 (3.5)	17 (5.0)	7 (2.0)	21 (3.6)	6 (2.3)	4 (2.3)	73 (7.5)	5 (2.9)	

Abbreviations: NA, not applicable; NE, New England.

^a^ Other race includes African; Alaskan Native; Asian, Pacific Islander, Native Hawaiian; mixed race; and other race.

^b^ Wisconsin did not collect data regarding sexual orientation.

**Table 2.** Distribution of Drug-Related HIV Transmission Behaviors Across Characteristics

Characteristic	Behaviors reported for past 30 d							
	Drug injection				Syringe sharing			
	Total, No.	No, No. (%)^a^	Yes, No. (%)^a^	*P* value	No.	0, No. (%)^a^	≥1 No. (%)^a^	*P* value
Overall	3046	459 (15.1)	2587 (84.9)^b^	NA	2433	1417 (58.2)	1016 (41.8)	NA
Age	3046	459 (15.1)	2587 (84.9)		2433	1417 (58.2)	1016 (41.8)	
Older	1626	285 (17.5)	1341 (82.5)	<.001	1261	792 (62.8)	469 (37.2)	<.001
Younger	1420	174 (12.3)	1246 (87.7)		1172	625 (53.3)	547 (46.7)	
Race	3043	459 (15.1)	2854 (84.9)		2403	1415 (58.2)	1015 (41.8)	
American Indian	225	16 (7.1)	209 (92.9)	<.001	185	106 (57.3)	70 (42.7)	.53
Black	96	27 (28.1)	69 (71.9)		66	33 (50.0)	33 (50.0)	
White	2574	386 (15.0)	2188 (85.0)		2070	1210 (58.5)	860 (41.5)	
Other^c^	148	30 (20.3)	118 (79.7)		109	66 (60.6)	43 (39.4)	
Gender identity	3044	459 (15.1)	2585 (84.9)		2432	1416 (58.2)	1016 (41.8)	
Men	1735	247 (14.2)	1488 (85.8)	.30	1387	837 (60.4)	550 (39.6)	.05
Women	1293	210 (16.2)	1083 (83.8)		1031	571 (55.4)	460 (44.6)	
Other	16	2 (12.5)	14 (87.5)		14	8 (57.1)	6 (42.9)	
Sexual orientation	2038	451 (22.1)	1587 (77.9)		1579	868 (55.0)	711 (45.0)	
Heterosexual	1769	400 (22.6)	1369 (77.4)	.12	1361	781 (57.4)	580 (42.6)	<.001
Gay or lesbian	40	10 (25.0)	30 (75.0)		30	17 (56.7)	13 (43.3)	
Bisexual	219	37 (16.9)	182 (83.1)		182	66 (36.3)	116 (63.7)	
Other	10	4 (40.0)	6 (60.0)		6	4 (66.7)	2 (33.3)	
Partnership status	2877	437 (15.2)	2440 (84.8)		2318	1345 (58.0)	973 (42.0)	
Partnered	905	156 (17.2)	749 (82.8)	.04	709	406 (57.3)	303 (42.7)	.65
Unpartnered	1972	281 (14.2)	1691 (85.8)		1609	939 (58.4)	670 (41.6)	
Drug of choice	3031	459 (15.1)	2572 (84.9)		2425	1411 (58.2)	1014 (41.8)	
Opioids	1636	294 (18.0)	1342 (82.0)	<.001	1292	700 (54.2)	592 (45.8)	<.001
Stimulants	1256	141 (11.2)	1115 (88.8)		1027	644 (62.7)	383 (37.3)	
Other	139	24 (17.3)	115 (82.7)		106	67 (63.2)	39 (36.8)	
Site, No. (%) range	3046	21 (12.1) [OR] to 93 (27.5) [KY]	245 (72.5) [KY] to 153 (87.9) [OR]	<.001	2433	82 (40.0) [OH] to 104 (68.4) [OR]	48 (31.6) [OR] to 123 (60.0) [OH]	<.001

Abbreviation: NA, not applicable.

^a^ Percentages are row percentages (per characteristic category).

^b^ Injection was required for Wisconsin participation, so past injection was 100% for Wisconsin.

^c^ Other includes African; Alaskan Native; Asian, Pacific Islander, or Native Hawaiian; mixed race; and other race.

**Table 3.** Distribution of HIV Transmission Behaviors Related to No. Sex Partners Across Characteristics

Characteristic	Behaviors reported for past 30 d															
	Sex with women								Sex with men							
	Among men^a^				Among women^b^				Among women^a^				Among men^b^			
	Total, No.	0-1, No. (%)^c^	≥2, No. (%)^c^	*P* value	No.	0, No. (%)^c^	≥1, No. (%)^c^	*P* value	No.	0-1, No. (%)^c^	≥2, No. (%)^c^	*P* value	No.	0, No. (%)^c^	≥1, No. (%)^c^	*P* value
Overall	1330	980 (73.7)	350 (26.3)	NA	1040	932 (89.6)	108 (10.4)	NA	1032	787 (76.3)	245 (23.7)	NA	1178	1085 (92.1)	93 (7.9)	NA
Age	1330	980 (73.7)	350 (26.3)		1040	932 (89.6)	108 (10.4)		1032	787 (76.3)	245 (23.7)		1178	1085 (92.1)	93 (7.9)	
Older	753	580 (77.0)	173 (23.0)	.002	555	514 (92.6)	41 (7.4)	<.001	551	450 (81.7)	101 (18.3)	<.001	666	615 (92.3)	51 (7.7)	.75
Younger	577	400 (69.3)	177 (30.7)		485	418 (86.2)	67 (13.8)		481	337 (70.1)	144 (29.9)		512	470 (91.8)	42 (8.2)	
Race	1330	980 (73.7)	350 (26.3)		1038	930 (89.6)	108 (10.4)		1030	785 (76.2)	245 (23.8)		1178	1085 (92.1)	93 (7.9)	
American Indian	66	40 (60.6)	26 (39.4)	.02	81	68 (84.0)	13 (16.0)	.004	80	57 (71.3)	23 (28.7)	.68	65	62 (95.4)	3 (4.6)	.52
Black	51	34 (66.7)	17 (33.3)		20	14 (70.0)	6 (30.0)		19	14 (73.7)	5 (26.3)		53	47 (88.7)	6 (11.3)	
White	1143	859 (75.2)	284 (24.8)		897	814 (90.7)	83 (9.3)		891	682 (76.5)	209 (23.5)		991	911 (91.9)	80 (8.1)	
Other^d^	70	47 (67.1)	23 (32.9)		40	34 (85.0)	6 (15.0)		40	32 (80.0)	8 (20.0)		69	65 (94.2)	4 (5.8)	
Gender identity																
Men	NA	NA	NA	NA	NA	NA	NA	NA	NA	NA	NA	NA	NA	NA	NA	NA
Women	NA	NA	NA		NA	NA	NA		NA	NA	NA		NA	NA	NA	
Other	NA	NA	NA		NA	NA	NA		NA	NA	NA		NA	NA	NA	
Sexual orientation	1140	849 (74.5)	291 (25.5)		888	807 (90.9)	81 (9.1)		886	682 (77.0)	204 (23.0)		993	945 (95.2)	48 (4.8)	
Heterosexual	1086	809 (74.5)	277 (25.5)	.11	681	659 (96.8)	22 (3.2)	<.001	681	544 (79.9)	137 (20.1)	<.001	938	920 (98.1)	18 (1.9)	<.001
Gay or lesbian	20	18 (90.0)	2 (10.0)		18	6 (33.3)	12 (66.7)		15	14 (93.3)	1 (6.7)		20	5 (25.0)	15 (75.0)	
Bisexual	31	21 (67.7)	10 (32.3)		182	136 (74.7)	46 (25.3)		183	117 (63.9)	66 (36.1)		32	18 (56.3)	14 (43.8)	
Other	3	1 (33.3)	2 (66.7)		7	6 (85.7)	1 (14.3)		7	7 (100)	0		3	2 (66.7)	1 (33.3)	
Partnership status	1251	933 (74.6)	318 (25.4)		1015	916 (90.2)	99 (9.8)		1009	771 (76.4)	238 (23.6)		1102	1019 (92.5)	83 (7.5)	
Partnered	352	300 (85.2)	52 (14.8)	<.001	350	317 (90.6)	33 (9.4)	.83	347	296 (85.3)	51 (14.7)	<.001	287	256 (89.2)	31 (10.8)	.02
Unpartnered	899	633 (70.4)	266 (29.6)		665	599 (90.1)	66 (9.9)		662	475 (71.8)	187 (28.2)		815	763 (93.6)	53 (6.4)	
Drug of choice	1330	980 (73.7)	350 (26.3)		1037	931 (89.8)	106 (10.2)		1030	785 (76.2)	245 (23.8)		1178	1085 (92.1)	93 (7.9)	
Opioids	767	588 (76.7)	179 (23.3)	.01	605	544 (89.9)	61 (10.1)	.34	602	472 (78.4)	130 (21.6)	.14	666	631 (94.7)	35 (5.3)	<.001
Stimulants	510	353 (69.2)	157 (30.8)		398	359 (90.2)	38 (9.8)		394	289 (73.4)	105 (26.6)		466	412 (88.4)	54 (11.6)	
Other	53	39 (73.6)	14 (26.4)		34	28 (82.4)	6 (17.6)		34	24 (70.6)	10 (29.4)		46	42 (91.3)	4 (8.7)	
Site, No. (%) range	1330	120 (65.9) [NC] to 161 (83.4) [KY]	32(16.6) [KY] to 62 (34.1) [NC]	.004	1040	120 (82.8) [WI] to 71 (97.3) [OR]	2 (2.7) [OR] to 25 (17.2) [WI]	.005	1032	98 (70.5) [WI] to 118 (84.3) [KY]	22 (15.7) [KY] to 41 (29.5) [WI]	.15	1178	133 (74.7) [WI] to 96 (97.0) [OR]	3 (3.0) [OR] to 45 (25.3) [WI]	<.001

Abbreviation: NA, not applicable.

^a^ Increased risk was assumed for more than 1 opposite-sex partner in the past 30 days.

^b^ Increased risk assumed for 1 or more same-gender partners in the past 30 days.

^c^ Percentages are row percentages (per characteristic category).

^d^ Other includes African; Alaskan Native; Asian, Pacific Islander, or Native Hawaiian; mixed race; and other race.

**Table 4.** Distribution of HIV Transmission Behaviors Related to Other Sex Behaviors Across Characteristics

Characteristic	Behaviors reported for past 30 d															
	Vaginal or anal sex				Transactional sex				Condomless sex							
									Overall				With someone who injects drugs			
	Total, No.	No, No. (%)^a^	Yes, No. (%)^a^	*P* value	No.	0, No. (%)^a^	≥1 No. (%)^a^	*P* value	No.	0, No. (%)^a^	≥1, No. (%)^a^	*P* value	No.	0, No. (%)^a^	≥1, No. (%)^a^	*P* value
Overall	2377	633 (26.6)	1744 (73.4)	NA	1799	1569 (87.2)	230 (12.5)	NA	1757	351 (20.0)	1406 (80.0)	NA	1391	487 (35.0)	904 (65.0)	NA
Age	2377	633 (26.6)	1744 (73.4)		1799	1569 (87.2)	230 (12.8)		1757	351 (20.0)	1406 (80.0)		1391	487 (35.0)	904 (65.0)	
Older	1306	432 (33.1)	874 (66.9)	<.001	906	792 (87.4)	114 (12.6)	.83	893	204 (22.8)	689 (77.2)	.002	682	235 (34.5)	447 (65.5)	.69
Younger	1071	201 (18.8)	870 (81.2)		893	777 (87.0)	116 (13.0)		864	147 (17.0)	717 (83.0)		709	252 (35.5)	457 (64.5)	
Race	2375	631 (26.6)	1744 (73.4)		1798	1568 (87.2)	230 (12.8)		1756	350 (19.9)	1406 (80.1)		1391	487 (35.0)	904 (65.0)	
American Indian	147	38 (25.9)	109 (74.1)	.83	115	105 (91.3)	10 (8.7)	.01	111	25 (22.5)	86 (77.5)	.32	86	28 (32.6)	58 (67.4)	.07
Black	73	16 (21.9)	57 (78.1)		57	42 (73.7)	15 (26.3)		57	15 (26.3)	42 (73.7)		42	20 (47.6)	22 (52.4)	
White	2043	547 (26.8)	1496 (73.2)		1543	1348 (87.4)	195 (12.6)		1507	290 (19.2)	1217 (80.8)		1202	425 (35.4)	777 (64.6)	
Other^b^	112	30 (26.8)	82 (73.2)		83	73 (88.0)	10 (12.0)		81	20 (24.7)	61 (75.3)		61	14 (23.0)	47 (77.0)	
Gender identity	2376	632 (26.6)	1744 (73.4)		1798	1568 (87.2)	230 (12.8)		1756	350 (19.9)	1406 (80.1)		1391	487 (35.0)	904 (65.0)	
Men	1332	377 (28.3)	955 (71.7)	.09	989	893 (90.3)	96 (9.7)	<.001	969	212 (21.9)	757 (78.1)	.08	746	267 (35.8)	479 (64.2)	.78
Women	1037	254 (24.5)	783 (75.5)		803	672 (83.7)	131 (16.3)		781	137 (17.5)	644 (82.5)		640	218 (34.1)	422 (65.9)	
Other	7	1 14.3	6 (85.7)		6	3 (50.0)	3 (50.0)		6	1 (16.7)	5 (83.3)		5	2 (40.0)	3 (60.0)	
Sexual orientation	2035	577 (28.4)	1458 (71.6)		1459	1281 (87.8)	178 (12.2)		1449	264 (18.2)	1185 (81.8)		1183	415 (35.1)	768 (64.9)	
Heterosexual	1769	518 (29.3)	1251 (70.7)	.02	1253	1128 (90.0)	125 (10.0)	<.001	1247	236 (18.9)	1011 (81.1)	.21	1009	370 (36.7)	639 (63.3)	.049
Gay or lesbian	39	9 (23.1)	30 (76.9)		30	25 (83.3)	5 (16.7)		29	6 (20.7)	23 (79.3)		23	7 (30.4)	16 (69.6)	
Bisexual	217	45 (20.7)	172 (79.3)		172	125 (72.7)	47 (27.3)		169	22 (13.0)	147 (87.0)		147	37 (25.2)	110 (74.8)	
Other	10	5 (50.0)	5 (50.0)		4	3 (75.0)	1 (25.0)		4	0	4 (100)		4	1 (25.0)	3 (75.0)	
Partnership status	2273	608 (26.7)	1665 (73.3)		1713	1498 (87.4)	215 (12.6)		1674	320 (19.1)	1354 (80.9)		1343	468 (34.8)	875 (65.2)	
Partnered	706	121 (17.1)	585 (82.9)	<.001	592	542 (91.6)	50 (8.4)	<.001	576	73 (12.7)	503 (87.3)	<.001	501	176 (35.1)	325 (64.9)	.91
Unpartnered	1567	487 (31.1)	1080 (68.9)		1121	956 (85.3)	165 (14.7)		1098	247 (22.5)	851 (77.5)		842	292 (34.7)	550 (65.3)	
Drug of choice	2374	633 (26.7)	1741 (73.3)		1796	1567 (87.2)	229 (12.8)		1755	351 (20.0)	1404 (80.0)		1389	487 (35.1)	902 (64.9)	
Opioids	1374	367 (26.7)	1007 (73.3)	.99	1025	901 (87.9)	124 (12.1)	.39	1015	201 (19.8)	814 (80.2)	.92	810	312 (38.5)	498 (61.5)	<.001
Stimulants	912	242 (26.5)	670 (73.5)		700	602 (86.0)	98 (14.0)		671	135 (20.1)	536 (79.9)		527	151 (28.7)	376 (71.3)	
Other	88	24 (27.3)	64 (72.7)		71	64 (90.1)	7 (9.9)		69	15 (21.7)	54 (78.3)		52	24 (46.2)	28 (53.8)	
Site, No. (%) range	2377	52 (15.9) [WI] to 78 (45.1) [OR]	95 (54.9) [OR] to 276 (84.1) [WI]	<.001	1799	149 (78.4) [OH] to 261 (96.0) [KY]	11 (4.0) [KY] to 41 (21.6) [OH]	<.001	1757	7 (7.6) [OR] to 82 (27.6) [WI]	215 (72.4) [WI] to 85 (92.4) [OR]	<.001	1391	21 (25.0) [OR) ] to 104 (44.4) [KY]	130 (55.6) [KY] to 63 (75.0) [OR]	<.001

^a^ Percentages are row percentages (per characteristic category).

^b^ Other includes African; Alaskan Native; Asian, Pacific Islander, or Native Hawaiian; mixed race; and other race.

**Table 5.** Multivariable Analysis of Association of Participant Characteristics With Past 30-d Behaviors

Characteristic	Behavior, aOR (95% CI)									
	Drug injection^a^	Syringe sharing	Vaginal or anal sex	Multiple women partners		Multiple men partners		Transactional sex	Condomless sex	
				≥2 Among men	≥1 Among women	≥2 Among women	≥1 Among men		Overall	With someone who injects drugs
Age										
Older	1 [Reference]	1 [Reference]	1 [Reference]	1 [Reference]	1 [Reference]	1 [Reference]	1 [Reference]	1 [Reference]	1 [Reference]	1 [Reference]
Younger	1.36 (1.08-1.72)	1.49 (1.20-1.85)	2.17 (1.75-2.71)	1.50 (1.12-2.01)	1.81 (1.02-3.20)	2.00 (1.41-2.84)	1.59 (0.70-3.61)	0.95 (0.67-1.35)	1.36 (1.01-1.82)	0.94 (0.73-1.21)
Race										
American Indian	1.13 (0.61-2.06)	0.87 (0.55-1.39)	0.86 (0.53-1.38)	1.50 (0.76-2.93)	1.40 (0.52-3.76)	1.20 (0.63-2.29)	NA^b^	0.61 (0.24-1.52)	0.83 (0.42-1.64)	0.98 (0.53-1.81)
Black	0.33 (0.19-0.59)	1.00 (0.47-2.13)	1.12 (0.59-2.11)	1.04 (0.47-2.33)	4.21 (0.91-19.40)	1.14 (0.29-4.49)	NA^b^	1.44 (0.57-3.66)	0.49 (0.23-1.02)	0.59 (0.26-1.35)
White	1 [Reference]	1 [Reference]	1 [Reference]	1 [Reference]	1 [Reference]	1 [Reference]	1 [Reference]	1 [Reference]	1 [Reference]	1 [Reference]
Other^c^	0.56 (0.34-0.90)	0.59 (0.34-1.02)	0.91 (0.57-1.47)	1.14 (0.61-2.11)	1.04 (0.30-3.60)	0.65 (0.26-1.64)	NA^b^	0.81 (0.35-1.87)	0.52 (0.28-0.95)	1.53 (0.75-3.13)
Gender										
Men	1 [Reference]	1 [Reference]	1 [Reference]	NA	NA	NA	NA	1 [Reference]	1 [Reference]	1 [Reference]
Women	0.77 (0.61-0.97)	0.93 (0.75-1.17)	1.04 (0.84-1.29)	NA	NA	NA	NA	1.46 (1.01-2.12)	1.05 (0.78-1.41)	0.97 (0.74-1.26)
Other	0.28 (0.04-2.23)	NA^b^	0.63 (0.06-6.58)	NA	NA	NA	NA	3.27 (0.24-43.97)	0.38 (0.03-5.36)	0.27 (0.02-4.75)
Sexual orientation										
Heterosexual	1 [Reference]	1 [Reference]	1 [Reference]	1 [Reference]	1 [Reference]	1 [Reference]	1 [Reference]	1 [Reference]	1 [Reference]	1 [Reference]
Gay or lesbian	0.90 (0.42-1.93)	1.11 (0.51-2.38)	1.43 (0.65-3.15)	0.32 (0.07-1.44)	64.22 (20.77-198.6)	0.26 (0.03-2.07)	300.6 (69.43-1300)	2.31 (0.83-6.42)	0.77 (0.30-1.97)	1.25 (0.50-3.17)
Bisexual	1.48 (0.99-2.22)	2.27 (1.60-3.23)	1.46 (1.02-2.14)	1.19 (0.52-2.73)	8.90 (4.98-15.90)	1.81 (1.23-2.65)	44.60 (16.21-122.7)	2.59 (1.67-4.03)	1.64 (0.98-2.75)	1.60 (1.04-2.46)
Other	0.46 (0.12-1.79)	0.73 (0.11-4.68)	0.52 (0.13-2.05)	6.33 (0.56-71.99)	5.04 (0.53-48.32)	NA^b^	55.53 (2.97-1039)	2.47 (0.24-25.90)	NA^b^	1.87 (0.19-18.64)
Partnership status										
Partnered	1 [Reference]	1 [Reference]	1 [Reference]	1 [Reference]	1 [Reference]	1 [Reference]	1 [Reference]	1 [Reference]	1 [Reference]	1 [Reference]
Unpartnered	1.37 (1.08-1.74)	0.82 (0.65-1.04)	0.54 (0.43-0.69)	2.37 (1.63-2.44)	1.10 (0.61-1.99)	2.22 (1.50-3.28)	0.44 (0.17-1.14)	1.71 (1.15-2.55)	0.61 (0.44-0.84)	1.01 (0.78-1.32)
Drug of choice										
Opioids	1 [Reference]	1 [Reference]	1 [Reference]	1 [Reference]	1 [Reference]	1 [Reference]	1 [Reference]	1 [Reference]	1 [Reference]	1 [Reference]
Stimulants	1.14 (0.89-1.47)	0.89 (0.71-1.12)	0.95 (0.76-1.19)	1.52 (1.11-2.06)	0.86 (0.47-1.57)	1.49 (1.04-2.14)	1.77 (0.73-4.28)	1.23 (0.84-1.79)	1.05 (0.76-1.44)	1.45 (1.09-1.93)
Other	0.53 (0.31-0.91)	1.21 (0.63-2.33)	1.04 (0.58-1.88)	1.52 (0.69-3.33)	1.31 (0.27-6.44)	1.66 (0.65-4.24)	1.02 (0.06-16.27)	0.81 (0.27-2.38)	1.21 (0.52-2.80)	0.65 (0.33-1.26)
Site										
IL	1 [Reference]	1 [Reference]	1 [Reference]	1 [Reference]	1 [Reference]	1 [Reference]	1 [Reference]	1 [Reference]	1 [Reference]	1 [Reference]
KY	0.89 (0.57-1.37)	1.23 (0.78-2.01)	1.32 (0.84-2.07)	0.51 (0.28-0.94)	0.47 (0.12-1.86)	0.63 (0.30-1.32)	0.56 (0.07-4.51)	0.50 (0.21-1.19)	1.24 (0.68-2.27)	0.74 (0.44-1.23)
NC	1.93 (1.17-3.17)	1.57 (0.98-2.51)	0.99 (0.63-1.56)	1.00 (0.56-1.79)	1.29 (0.44-3.85)	0.98 (0.48-1.97)	0.59 (0.11-3.13)	1.11 (0.52-2.36)	1.18 (0.64-2.20)	1.49 (0.86-2.59)
NE	0.90 (0.59-1.36)	1.73 (1.11-2.71)	0.73 (0.49-1.10)	0.73 (0.42-1.25)	1.17 (0.42-3.27)	0.87 (0.45-1.70)	1.18 (0.28-4.92)	1.56 (0.78-3.12)	0.72 (0.42-1.25)	0.85 (0.51-1.40)
OH	1.49 (0.92-2.42)	3.01 (1.85-4.91)	1.06 (0.67-1.67)	0.78 (0.42-1.46)	2.27 (0.76-6.75)	1.38 (0.68-2.81)	1.42 (0.29-6.99)	2.65 (1.29-5.41)	0.64 (0.36-1.15)	1.59 (0.89-2.84)
OR	2.39 (1.31-4.34)	1.03 (0.61-1.74)	0.41 (0.26-0.66)	0.50 (0.25-1.01)	0.38 (0.07-2.13)	0.83 (0.37-1.87)	0.25 (0.04-1.67)	1.25 (0.51-3.08)	2.51 (1.01-6.27)	1.63 (0.83-3.20)
WV	1.27 (0.76-2.13)	2.28 (1.35-3.86)	0.70 (0.43-1.13)	1.15 (0.62-2.15)	0.84 (0.21-3.33)	0.54 (0.22-1.33)	0.81 (0.14-4.82)	2.10 (0.91-4.24)	1.19 (0.60-2.38)	1.07 (0.58-1.96)

Abbreviations: aOR, adjusted odds ratio; NE, New England; NA, not applicable.

^a^ Wisconsin data are not included in the full models given that injection was required for participation (past injection = 100%), and sexual orientation data were not collected.

^b^ Not able to calculate (infinite).

^c^ Other includes African; Alaskan Native; Asian, Pacific Islander, or Native Hawaiian; mixed race; and other race.

## Results

### Participants

A total of 3048 PWUD (mean [SD] age, 36.1 [10.3] years; 225 American Indian [7.4%], 96 Black [3.2%], and 2576 White [84.5%] among 3045 with responses; and 1737 men [57.0%] among 3046 with responses) completed surveys, of whom 16 of 2610 respondents with responses (0.61%) were positive for HIV by rapid assay. Most participants were heterosexual (1771 individuals [86.8%] among 2040 with responses) and unpartnered (1974 individuals [68.6%] among 2879 with responses) (Table 1). Among 3033 participants with responses, opioids, stimulants, and other drugs were reported as the drug of choice by 1636 individuals (53.9%), 1258 individuals (41.5%), and 139 individuals (4.6%), respectively. Among 3033 individuals with responses, the most commonly reported drugs of choice were heroin (1146 individuals [37.8%]), methamphetamine or amphetamine (1070 individuals [35.3%]), opioid painkillers (293 individuals [9.7%]), cocaine or crack (188 individuals [6.2%]), and street fentanyl or carfentanil powder (67 individuals [2.2%]). Most participants reported recent injection (2587 of 3046 individuals [84.9%] with responses) and condomless sex (1406 of 1757 individuals [80.0%] with responses), among whom 904 of 1391 individuals (65.0%) with responses indicated that it occurred with people who inject drugs. Syringe sharing (1016 of 2433 individuals [41.8%] with responses) and transactional sex (230 of 1799 individuals [12.8%] with responses) were reported less frequently. The proportion of participants from each of 8 sites ranged from 173 individuals (5.7%) at Illinois to 991 individuals (32.5%) at Wisconsin. There was pervasive variation of exposures (ie, participant characteristics; eg, mean [SD] age range, 34.5 [10.0] years in Wisconsin to 39.7 [11.0] years in Illinois; *P* < .001) and outcomes (ie, behaviors; eg, transactional sex range, 11 of 272 responses [4.0%] in Kentucky to 41 of 190 responses [21.6%] in Ohio with data; *P* < .001) across study sites.

### Drug-Related Behaviors

The frequency of transmission behaviors associated with drug use (past 30-day injection and syringe sharing) varied significantly by age (eg, injection: 1246 of 1420 younger participants [87.7%] vs 1341 of 1626 older participants [82.5%] with responses), drug of choice (eg, injection: 1342 of 1636 participants using opioids [82.0%] vs 1115 of 1256 participants using stimulants [88.8%] vs 115 of 139 participants using other drugs [82.7%] with responses), and site (eg, range for injection: 245 of 338 participants [72.5%] in Kentucky to 153 of 174 participants in [87.9%] in Oregon with responses) (all *P* < .001) (Table 2). Furthermore, injection varied by race (209 of 225 American Indian participants [92.9%], 69 of 96 Black participants [71.9%], 2188 of 2574 White participants [85.0%], and 118 of 148 participants with other race [79.7%] with responses; *P* < .001) and partnership status (749 of 905 partnered [82.8%] and 1691 of 1972 unpartnered [85.8%] participants with responses; *P* = .02), and syringe sharing varied by sexual orientation (580 of 1361 heterosexual individuals [42.6%], 13 of 30 gay or lesbian individuals [43.3%], 116 of 182 bisexual individuals [63.7%], and 2 of 6 individuals with other orientation [33.3%] with responses; *P* < .001). In univariate modeling, several factors were associated with drug injection (eg, younger age: OR, 1.52; 95% CI, 1.24-1.87) and syringe sharing (eg, bisexual orientation: OR, 2.37; 95% CI, 1.72-2.23) (eTable in Supplement 1). In multivariable modeling, younger age (aOR, 1.36; 95% CI, 1.08-1.72), being single (aOR, 1.37; 95% CI, 1.08-1.74), and some sites were associated with recent injection, while younger age (aOR, 1.49; 95% CI,1.20-1.85), bisexual orientation (aOR vs heterosexual orientation, 2.27; 95% CI, 1.60-3.23), and some sites were associated with recent syringe sharing (Table 5).

### Sexual Behaviors

The frequency of transmission behaviors associated with sexual activity (any vaginal or anal sex, number of opposite- and same-gender partners, transactional sex, and condomless sex) exhibited substantial variability across characteristics, with 7 behaviors varying by site (eg, vaginal or anal sex: range, 95 of 173 participants [54.9%] in Oregon to 276 of 328 participants [84.1%] in Wisconsin; *P* < .001), 6 behaviors varying by sexual orientation (eg, transactional sex: 125 of 1253 heterosexual participants [10.0%], 5 of 30 gay or lesbian participants [16.7%], 47 of 172 bisexual participants [27.3%], 1 of 4 participants with other orientation [25.0%] with responses; *P* < .001) and partnership status (eg, condomless sex: 503 of 576 partnered [87.3%] and 851 of 1098 unpartnered [77.5%] individuals with responses; *P* < .001), 3 behaviors varying by race (eg, women partners among women: 13 of 81 American Indian women [16.0%], 6 of 20 Black women [30.0%], 83 of 897 White women [9.3%], and 6 of 40 women of other race [15.0%] with responses; *P* = .004), and 1 behavior (transactional sex) by gender identity (96 of 989 men [9.7%], 131 of 803 women [16.3%], and 3 of 6 transgender individuals [50.0%] with responses; *P* < .001) (Table 3 and Table 4). In multivariable modeling, increased odds of multiple opposite-gender women partners were observed among participants who were younger (aOR, 1.50; 95% CI, 1.12-2.01), were unpartnered (aOR, 2.37; 95% CI, 1.63-2.44), and reported stimulants as their drug of choice (aOR vs opioids1.52; 95% CI, 1.11-2.06) (Table 5). Increased odds of multiple opposite-sex men partners was observed for participants who were younger (aOR, 2.00; 95% CI, 1.41-2.84), bisexual (aOR vs heterosexual, 1.81; 95% CI, 1.23-2.65), and unpartnered (aOR, 2.22; 95% CI, 1.50-3.28) and those who reported stimulants as their drug of choice (aOR vs opioids, 1.49; 95% CI, 1.04-2.14). Transactional sex varied significantly by race, gender identity, sexual orientation, partnership status, and site (eg, race: 10 of 115 American Indian individuals [8.7%], 15 of 57 Black individuals [26.3%], 195 of 1543 White individuals [12.6%], and 10 of 80 individuals with other race [12.0%] with responses; *P* = .01). Increased odds of transactional sex were observed among participants who were women (aOR vs men, 1.46; 95% CI, 1.01-2.12), bisexual (aOR vs heterosexual, 2.59; 95% CI, 1.67-4.03), and unpartnered (aOR, 1.71; 95% CI, 1.15-2.55). Condomless sex with someone who injects drugs varied by sexual orientation, drug of choice, and site (eg, sexual orientation: 639 of 1009 heterosexual individuals [63.3%], 16 of 23 gay and lesbian individuals [69.6%], 110 of 147 bisexual individuals [74.8%], and 3 of 4 individuals with other orientation [75.0%] with responses; *P* = .049) (Table 4). Increased odds of condomless sex with someone who injects drugs were observed for participants who were bisexual (aOR vs heterosexual, 1.60; 95% CI, 1.04-2.46) and reported stimulants as their drug of choice (aOR vs opioids, 1.45; 95% CI, 1.09-1.93).

### Association of Location With Behaviors

There was substantial heterogeneity of participant characteristics and transmission-associated behaviors across sites. Age, race, gender identity, sexual orientation, partnership status, and drug of choice all varied by site (eg, age: mean [SD] range, 34.5 [10.0] years in Wisconsin to 39.7 [11.0] years in Illinois; *P* < .001), as did 9 of 10 behaviors (eg, syringe sharing: range, 48 of 152 participants [31.6%] in Oregon to 123 of 205 participants [60.0%] in Ohio with responses; *P* < .001) (Table 2, Table 3, and Table 4). Bivariate comparisons found multiple instances of different risk of transmission behaviors across sites, which were frequently substantiated in the multivariable model (eg, increased odds of syringe sharing in New England: aOR vs Illinois, 1.73; 95% CI, 1.11-2.71 and increased odds of transactional sex in Ohio: aOR vs Illinois, 2.65; 95% CI, 1.29-5.41) (Table 5).

### Association of Age With Behaviors

Similar to location, age also varied significantly across other participant characteristics (eg, gender identity: mean [SD] range, 29.3 [9.8] years among transgender individuals to 36.6 [10.5] years among men; *P* < .001), as well as drug-related and 5 of 8 sex-related behaviors (eg, drug injection: 1246 of 1420 younger individuals [87.7%] vs 1341 of 1626 older individuals [82.5%] with responses; *P* < .001) (Table 2, Table 3, and Table 4). Bivariate comparisons found increased odds for these 7 behaviors, which were each substantiated in the multivariable model for drug-related behaviors (recent injection: aOR for younger age, 1.36; 95% CI, 1.08-1.72; syringe sharing: aOR for younger age, 1.49; 95% CI, 1.20-1.85) and 5 sex-related behaviors (aOR for younger age ranging from 1.36; 95% CI, 1.01-1.82 for condomless sex to 2.17; 95% CI, 1.75-2.71 for vaginal or anal sex) (Table 5).

### Association of Sexual Orientation With Behaviors

Sexual orientation, specifically bisexual orientation, frequently varied across characteristics and was associated with increased risk of multiple behaviors. Sexual orientation varied by 3 characteristics (age, gender identity, and site; eg, for age: mean [SD] range, 33.6 [8.8] years among bisexual participants to 41.0 [14.7] years among transgender individuals; *P* < .001), syringe sharing (580 of 1361 heterosexual individuals [42.6%], 13 of 30 gay and lesbian individuals [43.3%], 116 of 182 bisexual individuals [63.7%], and 2 of 6 individuals with other orientation [33.3%] with responses; *P* < .001), and 6 sex-related behaviors (eg, condomless sex with someone who injects drugs) (Table 2, Table 3, and Table 4). Bivariate comparisons found increased odds for these 7 behaviors, which were substantiated in the multivariable model for syringe sharing (aOR for bisexual vs heterosexual orientation, 2.27; 95% CI, 1.60-3.23) and sex-related behaviors (aOR for bisexual vs heterosexual orientation ranging from 1.46; 95% CI,1.02-2.14 for vaginal or anal sex to 44.6; 95% CI, 16.21-122.7 for ≥1 men sexual partners among men) (Table 5).

## Discussion

In this cross-sectional study of a multistate convenience sample of rural PWUD, we found considerable and consistent heterogeneity of participant characteristics and HIV transmission–associated behaviors across regions. This may be associated, in part, with geographic differences in convenience sampling but likely also reflects the diversity of populations and their engagement or lack of engagement in locally available harm-reduction services. Proportions of participants with past 30-day experience with drug injection, vaginal or anal sex, multiple women partners, same-gender partners, transactional sex, condomless sex, and condomless sex with someone who injects drugs differed by site despite similar outreach and recruitment methods. These findings suggest that “rural PWUD” may not be a homogeneous construct for which uniform interventions can be expected to be universally effective in addressing behaviors associated with HIV transmission. While some interventions have a substantial evidence base and would be expected to have a degree of efficacy in nearly any location (eg, syringe service programs and medication for opioid use disorder), local populations should be explored to identify specific characteristics and behavior patterns that may be associated with more effective program implementation.

Our data demonstrate frequent engagement in behaviors associated with HIV transmission by rural PWUD. These findings suggest that interventions to reduce HIV risk by injection-related and sexual behavior should be tailored and implemented in rural communities. For example, syringe service programs and supervised injection sites are evidence-based ways to reduce HIV risk, but additional research is needed about how best to conduct these interventions in rural areas.^62^ Our data suggest core intervention components that should be part of all HIV risk reduction interventions in rural communities, such as ensuring that PWUD are educated about and provided the means, such as condoms and syringes, to address their specific risks. Given the relative paucity of clinical HIV prevention services in rural areas, further research should explore the utility of providing information regarding preexposure prophylaxis to PWUD and encouraging local clinicians to make preexposure prophylaxis accessible. Similarly, condom use should be encouraged with nonmonogamous or unfamiliar partners given that condomless sex was highly prevalent regardless of participant characteristics. Other HIV risk-reduction interventions may best be tailored to specific local PWUD characteristics, such as individuals of younger age, those with bisexual orientation, and those not in a partnered relationship, who all had increased odds of engaging in multiple risky sexual activities in our study. Interventions may also be developed to address specific behaviors, such as transactional sex, which varied by gender, race, and sexual orientation.

Adjusted models demonstrated that younger age, White race, gender identity as men, and being unpartnered (or single) were associated with recent drug injection. Younger age and bisexual orientation were also associated with syringe sharing. While our results regarding age, race, and gender were similar to those from previous studies, we could find no studies exploring increased risk of injection among individuals who were unpartnered or exploring increased risk of syringe sharing among those of bisexual orientation.^40,41,44,46^ Participant characteristics more frequently associated with risk of sexual transmission behaviors included younger age (for vaginal or anal sex and multiple partners) and bisexual orientation (for vaginal or anal sex, same-gender partners, transactional sex, and condomless sex with someone who injects drugs).

While we could find no studies exploring differences in sexual transmission behaviors among rural PWUD, data reported here suggest possible future research and interventions. For example, participants who were single (or unpartnered) were more likely to report multiple drug use and sexual transmission behaviors. Research should explore their socioeconomic environment to identify specific factors associated with risk and opportunities for intervention. PWUD of bisexual orientation were similarly more likely to report multiple transmission-associated behaviors. Here, existing HIV risk-reduction methods and messaging may be modified for specific inclusion of bisexual individuals while also exploring risks and strengths specifically associated with bisexual orientation.^63^

### Limitations

There are several limitations associated with this work. First, the cross-sectional nature of the surveys prevents studying temporal associations. Thus, we cannot know if some behaviors significantly preceded others and may be factors (eg, drug injection and transactional sex) associated with other outcomes. Second, peer recruiting may have led to recruitment of individuals similar to existing participants. While chain referral may work to overcome this limitation, many individuals did not recruit others or recruitment stopped at the second wave. Third, while the Rural Opioid Initiative was focused primarily on opioids (eligibility included any opioid misuse and any drug injection), stimulants were the drug of choice for many participants in this study and were associated with increased odds of injection and multiple sexual transmission behaviors; this was unsurprising given the long history of stimulants as “party drugs.”^64^ Given that rural PWUD would not have been eligible for inclusion if they used methamphetamine by noninjection routes and did not also misuse opioids, the true association of stimulant use with risky behaviors in this study was difficult to estimate. Fourth, there were multiple comparisons made, and because this is an exploratory and descriptive analysis, we did not perform adjustments for multiple comparisons. Thus, some more marginal associations may be due to random chance. Fifth, it should be noted that there were substantial differences in sample sizes and recruitment methods between sites, and differences by site may not reflect true, site-specific factors but rather the diversity of that sample. With an eye toward implementation and intervention, convenience samples identified at sites represented samples of PWUD who would reasonably be engaged through similar outreach efforts in interventions as were implemented during study recruitment. Overall, some major findings of this work (that rural PWUD frequently engaged in HIV transmission–associated behaviors and that relative frequencies and risks varied considerable across areas) may likely be generalizable to other rural areas in the US given that the study includes data from both coasts, the Midwest, and central Appalachia.

### Conclusions

Findings from this cross-sectional study suggest that the ROI may provide valuable data regarding rural HIV transmission–associated behaviors, especially at the intersection of injection drug use and sexual activity. While HIV infection was infrequently discovered, substantial proportions of participants engaged in both drug use and sexual behaviors that could be associated with the rapid spread of HIV. While the increase in syringe services programs has done much to reduce syringe sharing and introduce other harm-reduction measures, similar expansion of efforts should be used to address transmission risks associated with sexual behaviors and be tailored to locally relevant circumstances.

## Notes

Supplement 1.
eMethods.
**eTable.** Univariate Analysis of Association of Participant Characteristic With 30-d Behaviors

Supplement 2.
Data Sharing Statement
