# Supplementary material for: Variation in HIV Transmission Behaviors Among People Who Use Drugs in Rural US Communities
Source: JAMA Netw Open. 2023 Aug 21;6(8):e2330225. doi: 10.1001/jamanetworkopen.2023.30225 (PMC10442709; doi:10.1001/jamanetworkopen.2023.30225)
Supplement: Supplement 1. — eMethods. eTable. Univariate Analysis of Association of Participant Characteristic With 30-d Behaviors [file jamanetwopen-e2330225-s001.pdf]

Supplemental Online Content

Jenkins WD, Friedman SR, Hurt CB, et al. Variation in HIV transmission behaviors among people who use drugs in rural US communities. *JAMA Netw Open*. 2023;6(8):e2330225. doi:10.1001/jamanetworkopen.2023.30225

eMethods.
eTable. Univariate Analysis of Association of Participant Characteristic With 30-d Behaviors

This supplemental material has been provided by the authors to give readers additional information about their work.

© 2023 Jenkins WD et al. *JAMA Network Open.*

eMethods.

Site institutional review boards and software version numbers:

Site	IRB	Software
IL	#IRB17-1630	ACASI version 6.0 (Audio Computer-Assisted Self-Interview)
KY	#43520	interviewer-administration of Computer-Assisted Personal Interviews (CAPI) in QDS v4.0
NC	(Internal RTI IRB): CR00000959	ACASI version 6.0 (Audio Computer-Assisted Self-Interview)
NEng	#1094092	ACASI version 6.0 (Audio Computer-Assisted Self-Interview)
OH	#2017B0328	ACASI version 6.0 (Audio Computer-Assisted Self-Interview)
OR	#STUDY00017233	Computer Assisted Self-Interviews (CAPI) in REDCap 13.7.2
WV	#1801956569	ACASI version 6.0 (Audio Computer-Assisted Self-Interview)
WI	#2017-0866	Computer Assisted Self-Interviews (CAPI) in REDCap version 11.1.29

All studies administered a standardized survey at the enrollment interview; five studies administered it using a centrally-developed Audio Computer-Assisted Self-Interview (ACASI), two used Computer Assisted Self-Interviews (CASI) in REDCap, and one involved interviewer-administration of Computer-Assisted Personal Interviews (CAPI) in QDS (Questionnaire Development System™).

© 2023 Jenkins WD et al. *JAMA Network Open.*

eTable. Univariate Analysis of Association of Participant Characteristic With 30-d Behaviors

Characteristic	Behavior, OR (95% CI)									
	Drug injection	Syringe sharing	Vaginal or anal sex	Multiple women partners		Multiple men partners		Transactional sex	Condomless sex	
				≥2 Among men	≥1 Among women	≥2 Among women	≥1 Among men		Overall	With someone who injects drugs
Age										
Older	1 [Reference]	1 [Reference]	1 [Reference]	1 [Reference]	1 [Reference]	1 [Reference]	1 [Reference]	1 [Reference]	1 [Reference]	1 [Reference]
Younger	1.52 (1.24-1.87)	1.48 (1.26-1.74)	2.14 (1.77-2.59)	1.48 (1.16-1.90)	2.01 (1.33-3.03)	1.90 (1.42-2.55)	1.08 (0.70-1.65)	1.04 (0.79-1.37)	1.44 (1.14-1.83)	0.95 (0.77-1.19)
Race										
American Indian	2.30 (1.37-3.88)	1.05 (0.77-1.42)	1.05 (0.72-1.54)	1.97 (1.18-3.28)	1.88 (0.99-3.54)	1.32 (0.79-2.19)	0.55 (0.17-1.80)	0.66 (0.34-1.28)	0.82 (0.52-1.30)	1.13 (0.71-1.81)
Black	0.45 (0.29-0.71)	1.41 (0.86-2.30)	1.30 (0.74-2.29)	1.51 (0.83-2.75)	4.20 (1.57-11.23)	1.17 (0.42-3.27)	1.45 (0.60-3.50)	2.47 (1.34-4.54)	0.67 (0.37-1.22)	0.60 (0.33-1.12)
White	1 [Reference]	1 [Reference]	1[Reference]	1 [Reference]	1 [Reference]	1 [Reference]	1 [Reference]	1 [Reference]	1 [Reference]	1 [Reference]
Other[a]	0.69 (0.46-1.05)	0.92 (0.62-1.36)	1.00 (0.65-1.54)	1.48 (0.88-2.48)	1.73 (0.71-4.24)	0.82 (0.37-1.80)	0.70 (0.25-1.97)	0.95 (0.48-1.87)	0.73 (0.43-1.22)	1.84 (1.00-3.37)
Gender										
Men	1 [Reference]	1 [Reference]	1 [Reference]	NA	NA	NA	NA	1 [Reference]	1 [Reference]	1 [Reference]
Women	0.86 (0.70-1.05)	1.23 (1.04-1.44)	1.22 (1.01-1.46)	NA	NA	NA	NA	1.81 (1.37-2.40)	1.32 (1.04-1.67)	1.08 (0.87-1.35)
Other	1.16 (0.26-5.14)	1.14 (0.39-3.31)	2.37 (0.28-19.74)	NA	NA	NA	NA	9.30 (1.85-46.73)	1.40 (0.16-12.05)	0.84 (0.14 (5.04)
Sexual orientation										

© 2023 Jenkins WD et al. *JAMA Network Open.*

Heterosexual	1 [Reference]	1 [Reference]	1 [Reference]	1 [Reference]	1 [Reference]	1 [Reference]	1 [Reference]	1 [Reference]	1 [Reference]	1 [Reference]
Gay or lesbian	0.88 (0.43-1.81)	1.03 (0.50-2.14)	1.38 (0.65-2.93)	0.33 (0.08-1.41)	59.91 (20.59-174.3)	0.28 (0.04-2.18)	153.3 (50.31-467.3)	1.81 (0.68-4.80)	0.90 (0.36-2.22)	1.32 (0.54-3.25)
Bisexual	1.44 (0.99-2.08)	2.37 (1.72-3.26)	1.58 (1.12-2.23)	1.39 (0.65-2.99)	10.13 (5.90-17.40)	2.24 (1.57-3.20)	39.75 (17.16-92.07)	3.39 (2.31-4.98)	1.56 (0.98-2.50)	1.72 (1.16-2.55)
Other	0.44 (0.12-1.56)	0.67 (0.12-3.69)	0.41 (0.12-1.44)	5.84 (0.53-64.67)	4.99 (0.58-43.26)	NA[b]	25.56 (2.22-294.8)	3.01 (0.31-29.14)	NA[b]	1.74 (0.18-16.76)
Partnership status										
Partnered	1 [Reference]	1 [Reference]	1 [Reference]	1 [Reference]	1 [Reference]	1 [Reference]	1 [Reference]	1 [Reference]	1 [Reference]	1 [Reference]
Unpartnered	1.25 (1.01-1.55)	0.96 (0.80-1.14)	0.46 (0.37-0.57)	2.42 (1.75-3.36)	1.06 (0.68-1.64)	2.29 (1.62-3.22)	0.56 (0.35-0.90)	1.87 (1.34-2.61)	0.50 (0.38-0.66)	1.02 (0.81-1.29)
Drug of choice										
Opioids	1 [Reference]	1 [Reference]	1 [Reference]	1 [Reference]	1 [Reference]	1 [Reference]	1 [Reference]	1 [Reference]	1 [Reference]	1 [Reference]
Stimulants	1.73 (1.40-2.15)	0.70 (0.60-0.83)	1.01 (0.84-1.22)	1.46 (1.14-1.88)	0.97 (0.63-1.48)	1.32 (0.98-1.77)	2.36 (1.52-3.68)	1.18 (0.89-1.57)	0.98 (0.77-1.25)	1.56 (1.23-1.98)
Other	1.05 (0.66-1.66)	0.69 (0.46-1.04)	0.97 (0.60-1.58)	1.18 (0.63-2.22)	1.91 (0.76-4.80)	1.51 (0.71-3.24)	1.72 (0.58-5.06)	0.80 (0.36-1.77)	0.89 (0.49-1.61)	0.73 (0.42-1.28)
Site										
IL	1 [Reference]	1 [Reference]	1 [Reference]	1 [Reference]	1 [Reference]	1 [Reference]	1 [Reference]	1 [Reference]	1 [Reference]	1 [Reference]
KY	0.95 (0.63-1.44)	1.26 (0.80-1.99)	1.52 (0.99-2.34)	0.47 (0.27-0.84)	0.29 (0.09-0.93)	0.57 (0.28-1.15)	1.27 (0.35-4.57)	0.33 (0.15-0.76)	1.49 (0.85-2.62)	0.71 (0.44-1.15)
NC	2.12 (1.36-3.33)	1.56 (1.00-2.41)	1.17 (0.78-1.77)	1.23 (0.72-2.09)	1.10 (0.46-2.62)	1.08 (0.58-2.04)	0.52 (0.18-1.53)	0.82 (0.41-1.64)	1.16 (0.67-2.01)	1.56 (0.94-2.58)
NE	0.99 (0.68-1.46)	1.90 (1.25-2.88)	0.83 (0.57-1.22)	0.82 (0.50-1.34)	1.02 (0.44-2.36)	0.94 (0.51-1.72)	0.68 (0.27-1.70)	1.39 (0.75-2.58)	0.85 (0.51-1.41)	0.85 (0.53-1.34)
OH	1.44 (0.91-2.26)	3.04 (1.90-4.84)	1.04 (0.67-1.62)	0.80 (0.44-1.45)	1.07 (0.43-2.66)	1.14 (0.59-2.20)	0.65 (0.21-1.98)	2.18 (1.13-4.20)	0.66 (0.38-1.14)	1.57 (0.90-2.73)

© 2023 Jenkins WD et al. *JAMA Network Open.*

OR	2.64 (1.50-4.65)	0.93 (0.56-1.55)	0.45 (0.29-0.70)	0.57 (0.30-1.11)	0.23 (0.05-1.12)	0.91 (0.43-1.95)	0.41 (0.10-1.62)	1.06 (0.46-2.46)	2.86 (1.17-6.96)	1.71 (0.90-3.23)
WI	NA[c]	1.12 (0.75-1.68)	1.95 (1.25-3.06)	1.02 (0.60-1.75)	1.69 (0.72-3.97)	1.28 (0.67-2.44)	4.40 (1.90-10.19)	1.35 (0.72-2.56)	0.62 (0.37-1.03)	1.12 (0.68-1.85)
WV	1.34 (0.82-2.20)	2.24 (1.36-3.71)	0.70 (0.44-1.11)	1.09 (0.60-1.98)	0.81 (0.27-2.48)	0.61 (0.26-1.41)	0.50 (0.14-1.76)	1.59 (0.76-3.33)	1.18 (0.61-2.28)	1.02 (0.57-1.83)

Abbreviations: NA, not applicable; NE, New England; OR, odds ratio.

[a]Other includes African; Alaskan Native; Asian, Pacific Islander, or Native Hawaiian; mixed race; and other race.

[b]Not able to calculate (infinite).

[c]Injection was required for Wisconsin participation, so past injection was 100% for Wisconsin.

© 2023 Jenkins WD et al. *JAMA Network Open.*
