# Supplementary material for: Variation in HIV Transmission Behaviors Among People Who Use Drugs in Rural US Communities
Source: JAMA Netw Open. 2023 Aug 21;6(8):e2330225. doi: 10.1001/jamanetworkopen.2023.30225 (PMC10442709; doi:10.1001/jamanetworkopen.2023.30225)
Supplement: Supplement 2. — Data Sharing Statement [file jamanetwopen-e2330225-s002.pdf]

## Data Sharing Statement

Jenkins. Variation in HIV Transmission Behaviors Among People Who Use Drugs in Rural US Communities. *JAMA Netw Open*. Published online August 21, 2023. doi:10.1001/jamanetworkopen.2023.30225

**Data**
**Data available:** Yes
**Data types:** Deidentified participant data, Data dictionary
**How to access data:** The data from all sites were consolidated by a NIH/NIDA-funded Data Coordinating Center which has created a cleaned, de-identified data set (from which these analyses were performed). Investigators interested in accessing data sharing may contact the Data Coordinating Center at ruralopioids@uw.edu.
**When available:** beginning date: 06-01-2022

**Supporting Documents**
**Document types:** None

**Additional Information**
**Who can access the data:** Researchers whose proposed use of the data has been approved.
**Types of analyses:** Any analysis.
**Mechanisms of data availability:** After approval of a proposal.
